# Predictive ability of frailty scores in surgically managed patients with traumatic spinal injuries: a TQIP analysis

**DOI:** 10.1007/s00068-025-02775-0

**Published:** 2025-03-04

**Authors:** Maximilian Peter Forssten, Lovisa Ekestubbe, Yang Cao, Ahmad Mohammad Ismail, Ioannis Ioannidis, Babak Sarani, Shahin Mohseni

**Affiliations:** 1 Department of Orthopedic Surgery, Orebro University Hospital, Orebro, 701 85 Sweden; 2 School of Medical Sciences, Orebro University, Orebro, 702 81 Sweden; 3 Clinical Epidemiology and Biostatistics, School of Medical Sciences, Faculty of Medicine and Health, Orebro University, Orebro, 701 82 Sweden; 4 Center of Trauma and Critical Care, George Washington University, Washington, DC USA

**Keywords:** Traumatic spinal injury, Frailty, Orthopedic frailty score, Modified frailty index, Hospital frailty risk score, Johns Hopkins frailty indicator, Mortality, Morbidity

## Abstract

**Purpose:**

Frailty has gained recognition as a crucial determinant of patient outcomes following traumatic spinal injury (TSI), particularly due to its increasing incidence in elderly populations. The aim of the current investigation was therefore to compare the ability of several frailty scores to predict adverse outcomes in surgically managed isolated TSI patients without spinal cord injury.

**Methods:**

All adult patients (18 years or older) who suffered an isolated TSI due to blunt trauma, and required surgical management, were extracted from the 2013–2021 Trauma Quality Improvement Program database. The ability of the Orthopedic Frailty Score (OFS), the Hospital Frailty Risk Score (HFRS), the 11-factor (11-mFI) and 5-factor (5-mFI) modified frailty index, as well as the Johns Hopkins Frailty Indicator to predict adverse outcomes was compared based on the area under the receiver-operating characteristic curve (AUC). Subgroup analyses were also performed on patients who were ≥ 65 years old and those who were injured due to a ground-level fall (GLF).

**Results:**

A total of 39,449 patients were selected from the TQIP database. The 5-mFI and 11-mFI outperformed all other frailty scores when predicting in-hospital mortality (5-mFI AUC: 0.73) (11-mFI AUC: 0.73), any complication (5-mFI AUC: 0.65) (11-mFI AUC: 0.65), and FTR (5-mFI AUC: 0.75) (11-mFI AUC: 0.75). Among the 14,257 geriatric patients, however, the OFS demonstrated the highest predictive ability for in-hospital mortality (AUC: 0.65). The OFS (AUC: 0.64) also performed on the same level as both the 5-mFI (AUC: 0.63) and the 11-mFI (AUC: 0.63) when predicting FTR in this population. Among the 9616 patients who were injured due to a GLF, the OFS performed on par with the 5-mFI and 11-mFI when predicting in-hospital mortality and FTR.

**Conclusion:**

Simpler scores like the 5-factor modified Frailty Index and Orthopedic Frailty Score outperform or perform on par with more complicated frailty scores when predicting mortality, complications, and failure-to-rescue in surgically managed isolated traumatic spinal injury patients without spinal cord injury, particularly among geriatric patients and those injured in a GLF.

**Supplementary Information:**

The online version contains supplementary material available at 10.1007/s00068-025-02775-0.

## Introduction

Traumatic spinal injury (TSI) constitutes a considerable health burden, impacting almost 800,000 individuals worldwide annually and significantly contributing to trauma-related morbidity and mortality [1]. Frailty has gained recognition as a crucial determinant of patient outcomes following TSI, particularly due to its increasing incidence in elderly populations [2, 3]. Frailty, defined by a deterioration in the functioning of various physiological systems and heightened susceptibility to stressors, is linked to a greater risk of in-hospital mortality, complications, failure-to-rescue (FTR), as well as admission to the intensive care unit in TSI patients [3–5]. The utilization of tools such as frailty scores in clinical settings is crucial for identifying individuals at increased risk who may necessitate additional interventions, resources, and monitoring. By accurately identifying patients with elevated frailty, these scores could permit healthcare providers to make informed decisions regarding care intensity, resource allocation, and individualized treatment plans [6–10]. 

For patients undergoing surgical intervention for TSI, the variety of available frailty scoring systems presents a challenge for practitioners when deciding which to use in clinical practice. Currently, large-scale studies comparing these scores are lacking, leaving uncertainty about whether to favor simpler scores with fewer, easily accessible variables or more complex scores that offer greater detail. Standardizing the choice of scoring system could streamline assessments and enhance the consistency and quality of care provided to patients undergoing surgery for TSI. However, much focus has already been placed on patients with spinal cord injury (SCI) [11–13]. The aim of the current investigation was therefore to compare the ability of several frailty scores to predict adverse outcomes in surgically managed isolated TSI patients without SCI, with the hypothesis that more complicated models would result in diminishing returns.

## Methods

Data for the current investigation was retrieved from the 2013–2021 American College of Surgeons Trauma Quality Improvement Program (TQIP) database. Data abstracted including demographic and clinical characteristics as well as adverse outcomes. The current investigation adhered to the Strengthening the Reporting of Observational Studies in Epidemiology (STROBE) guidelines as well as the Declaration of Helsinki [14]. Due to the retrospective, anonymized nature of the dataset, the need for ethical approval was waived for the current study.

All adults (≥ 18 years old) who underwent surgical treatment for an isolated spinal injury resulting from a blunt trauma were included. An isolated spinal injury was defined as a spine AIS ≥ 2 and an AIS ≤ 1 in the remaining regions along with an International Classification of Disease-9 (ICD-9) or ICD-10 code consistent with a vertebral fracture. Surgical management was also identified based on recorded ICD-9 and ICD-10 codes [15]. Patients were excluded if they had a spine AIS of 6 since these injuries are generally not considered survivable.

### Calculating frailty scores

#### Orthopedic frailty score

The Orthopedic Frailty Score (OFS) was originally validated in hip fracture patients [6, 16], but has also been employed in patients with TSIs, where it has been found to be among the top predictors of adverse outcomes in this population [3, 15]. The OFS was determined based on the presence of five variables: an age ≥ 85 years old, institutionalization, non-independent functional status, congestive heart failure, and a history of malignancy. Patients received one point for each variable present, resulting in a maximum possible score of 5 [6]. 

#### Hospital frailty risk score

The Hospital Frailty Risk Score (HFRS) was calculated as the weighted sum of the 109 ICD-10 codes selected by Gilbert et al. Patients with an HFRS < 5 were categorized as low frailty risk, those with a score of 5–15 were categorized as intermediate frailty risk, while those with a score > 15 were considered to have a high frailty risk [7]. 

#### 11-Factor modified frailty index

The 11-factor modified Frailty Index (11-mFI) was calculated based on the presence of hypertension, peripheral vascular disease, angina pectoris, previous myocardial infarction, congestive heart failure, diabetes mellitus, stroke with neurologic deficit, transient ischemic attack or stroke without neurologic deficit, chronic obstructive pulmonary disease (COPD), non-independent functional status, and impaired sensorium [8, 17]. Pneumonia was not included in the calculation as it was a component of several of the outcomes in the current investigation. As each variable present increased the 11-mFI by 1 the maximum possible score was 11.

#### 5-Factor modified frailty index

The 5-factor modified Frailty Index (5-mFI) was calculated based on the presence of hypertension, congestive heart failure, diabetes mellitus, COPD, and non-independent functional status. Each variable present was worth one point with the maximum potential score being 5 [9]. 

#### Johns Hopkins frailty indicator

Patients were classified as either non-frail or frail, according to the Johns Hopkins Frailty Indicator, based on the presence of one of the frailty defining diagnoses. These diagnoses included profound visual impairment, senile dementia with delirium, senile dementia with delusional or depressive features, nutritional marasmus, other severe protein-calorie malnutrition, decubitus ulcer, fecal incontinence, continuous urinary leakage, incontinence without sensory awareness, feeding difficulties and mismanagement, abnormal loss of weight and underweight, inadequate material resources, inadequate housing, lack of housing, fall from wheelchair, fall on stairs or steps, abnormality of gait, or difficulty in walking [10, 18]. 

### Statistical analysis

With the purpose of describing the dataset, age was summarized as a median and interquartile range, while the remaining variables were presented as counts and percentages. The adverse outcomes investigated included in-hospital mortality, any complication, cardiovascular complications, venous thromboembolism, respiratory complications, infection, and failure-to-rescue (FTR).

Complications included were myocardial infarction, cardiac arrest with CPR, stroke, deep vein thrombosis (DVT), pulmonary embolism, acute respiratory distress syndrome (ARDS), acute kidney injury, urinary tract infection, pneumonia, surgical site infection, sepsis, decubitus ulcer, unplanned intubation, unplanned admission to the OR, and unplanned admission to the ICU. Cardiovascular complications included myocardial infarction, cardiac arrest, and stroke. Venous thromboembolism included DVT and pulmonary embolism. Respiratory complications included ARDS and pneumonia. Infections included urinary tract infection, pneumonia, surgical site infection, and sepsis. FTR was defined as in-hospital mortality subsequent to a complication.

The ability of the frailty scores to predict adverse outcomes was calculated based on the area under the receiver operating characteristic curve (AUC). To determine the AUC, a logistic regression model was fitted with the adverse outcome as the response variable and the frailty score (treated as a continuous variable) as the predictor, no other covariates were included in the model. In order to put the AUC into context, the sensitivity, specificity, and accuracy was also calculated at the threshold that maximized Youden’s index (sensitivity + specificity − 1). The confidence intervals (CIs) for the AUCs were calculated based on the variance of the AUC as defined by DeLong et al. [19], using the algorithm described by Sun and Xu [20]. The other CIs were determined using 2000 stratified bootstrap replicates.

These analyses were also repeated with a subgroup of patients consisting of those who were 65 years or older as well as a second subgroup consisting of patients who were injured as a result of a ground-level fall, as these populations are more likely to be frail [21, 22]. Statistical significance was defined as a two-sided *p*-value < 0.05. *P*-values were adjusted for multiple comparisons using the Holm-Bonferroni method. All analyses were performed in the software R 4.2.2 using the tidyverse, survey, parallel, and pROC packages [23]. 

## Results

After applying the inclusion and exclusion criteria, 39,449 adult patients remained who underwent surgery for an isolated TSI without SCI. The median age of the patient population was 56, with 50% of patients being between 37 and 71 years of age. The majority of patients were male (64.0%) and Non-Hispanic White (72.1%). Based on the OFS, 85.7% of the patients were classified as non-frail, 11.1% as pre-frail, and 3.2% as frail. Similarly, the HFRS categorized most patients (94.2%) as having a low frailty risk, with 5.7% at intermediate risk and only 0.1% at high risk. According to the 11-mFI, 53.8% of patients had no frailty indicators, while 25.4% had one, and 14.7% had two, with decreasing proportions as the number of frailty markers increased. The 5-mFI showed similar results, with 54.0% having no markers of frailty. The Johns Hopkins Frailty Indicator (JHFI) classified 93.0% of patients as non-frail and 7.0% as frail. The most common comorbidities consisted of hypertension (38.4%), diabetes mellitus (17.0%), major psychiatric illness (11.2%), alcohol use disorder (7.4%), and chronic obstructive pulmonary disease (7.1%) (Table 1).

**Table 1:** Demographics of patients with surgically managed isolated traumatic spinal injuries without spinal cord injury

	Total (*N* = 39,449)
Age, median [IQR]	56 [37–71]
Sex, n (%)	
Female	14,148 (35.9)
Male	25,243 (64.0)
Missing	58 (0.1)
Race, n (%)	
Non-Hispanic White	28,447 (72.1)
Hispanic or Latino	1,571 (4.0)
Black	3,219 (8.2)
Other	3,689 (9.4)
Missing	2,523 (6.4)
OFS, n (%)	
Non-frail (OFS 0)	33,806 (85.7)
Pre-frail (OFS 1)	4,386 (11.1)
Frail (OFS ≥ 2)	1,257 (3.2)
HFRS, n (%)	
Low frailty risk (HFRS < 5)	37,157 (94.2)
Intermediate frailty risk (HFRS 5–15)	2,264 (5.7)
High frailty risk (HFRS > 15)	28 (0.1)
11-mFI, n (%)	
0	21,221 (53.8)
1	10,035 (25.4)
2	5,792 (14.7)
3	1,831 (4.6)
4	461 (1.2)
5	103 (0.3)
6	6 (0.0)
5-mFI, n (%)	
0	21,293 (54.0)
1	10,168 (25.8)
2	5,848 (14.8)
3	1,701 (4.3)
4	391 (1.0)
5	48 (0.1)
JHFI, n (%)	
Non-frail (JHFI 0)	36,668 (93.0)
Frail (JHFI 1)	2,781 (7.0)
Hypertension, n (%)	15,145 (38.4)
History of angina, n (%)	61 (0.2)
Previous myocardial infarction, n (%)	353 (0.9)
Congestive heart failure, n (%)	1,615 (4.1)
History of peripheral vascular disease, n (%)	272 (0.7)
Cerebrovascular disease, n (%)	836 (2.1)
Impaired sensorium, n (%)	31 (0.1)
Dementia, n (%)	1,277 (3.2)
Non-independent functional status, n (%)	2,519 (6.4)
Institutionalized, n (%)	719 (1.8)
Currently receiving chemotherapy for cancer, n (%)	127 (0.3)
Metastatic cancer, n (%)	190 (0.5)
History of malignancy, n (%)	336 (0.9)
COPD, n (%)	2,801 (7.1)
Current smoker, n (%)	8,671 (22.0)
Chronic renal failure, n (%)	511 (1.3)
Diabetes mellitus, n (%)	6,691 (17.0)
Cirrhosis, n (%)	336 (0.9)
Coagulopathy, n (%)	1,072 (2.7)
Drug use disorder, n (%)	2,313 (5.9)
Alcohol use disorder, n (%)	2,908 (7.4)
Major psychiatric illness, n (%)	4,433 (11.2)
Advanced directive limiting care, n (%)	993 (2.5)

OFS, Orthopedic Frailty Score; HFRS, Hospital Frailty Risk Score; 11-mFI, 11-factor modified Frailty Index; 5-mFI, 5-factor modified Frailty Index; JHFI, Johns Hopkins Frailty Indicator; COPD, chronic obstructive pulmonary disease

As SCIs were excluded, only a minority suffered a severe spine injury (Spine AIS ≥ 3: 37.3%). Cervical spine injuries were most common (48.2%), followed by lumbar (35.5%) and thoracic (33.2%) injuries. Consequently, surgery involving the cervical spine was the most frequently performed (61.7%) (Table 2). 1.4% (*N* = 554) of patients died in the hospital, of these 63.0% (*N* = 349) of deaths occurred after a complication. 7.4% (*N* = 2,924) of patients suffered a complication; the most common were unplanned ICU admission (2.7%), unplanned intubation (1.9%), urinary tract infection (0.9%), and pneumonia (0.9%) (Table 3).

**Table 2:** Clinical characteristics of patients with surgically managed isolated traumatic spinal injuries without spinal cord injury

	Total (*N* = 39,449)
Injury Severity Score, n (%)	
0–9	31,352 (79.5)
10–15	6,924 (17.6)
16–25	1,011 (2.6)
26–75	162 (0.4)
Head AIS, n (%)	
Injury not present	34,212 (86.7)
1	5,237 (13.3)
Face AIS, n (%)	
Injury not present	33,658 (85.3)
1	5,791 (14.7)
Neck AIS, n (%)	
Injury not present	38,957 (98.8)
1	492 (1.2)
Spine AIS, n (%)	
2	24,731 (62.7)
3	14,009 (35.5)
4	470 (1.2)
5	239 (0.6)
Thorax AIS, n (%)	
Injury not present	36,823 (93.3)
1	2,626 (6.7)
Abdomen AIS, n (%)	
Injury not present	38,220 (96.9)
1	1,229 (3.1)
Upper extremity AIS, n (%)	
Injury not present	35,030 (88.8)
1	4,419 (11.2)
Lower extremity AIS, n (%)	
Injury not present	35,543 (90.1)
1	3,906 (9.9)
External/Other AIS, n (%)	
Injury not present	37,941 (96.2)
1	1,508 (3.8)
Level of spine injury, n (%)	
Cervical	19,009 (48.2)
Thoracic	13,101 (33.2)
Lumbar	13,986 (35.5)
Level of spine surgery, n (%)	
Cervical	24,334 (61.7)
Thoracic	22,985 (58.3)
Lumbar	18,998 (48.2)
Systolic blood pressure < 90 mmHg, n (%)	276 (0.7)
Missing	1,049 (2.7)
Pulse rate > 100 bpm, n (%)	6,019 (15.3)
Missing	1,056 (2.7)
Temperature < 35 °C, n (%)	148 (0.4)
Missing	3,232 (8.2)
Temperature ≥ 38 °C, n (%)	224 (0.6)
Missing	3,232 (8.2)
Saturation < 90%, n (%)	640 (1.6)
Missing	2,032 (5.2)
Respiratory rate > 20, n (%)	4,743 (12.0)
Missing	1,311 (3.3)
Respiratory rate < 20, n (%)	459 (1.2)
Missing	1,311 (3.3)
GCS on admission, n (%)	
Mild (GCS 14–15)	36,456 (92.4)
Moderate (GCS 9–13)	533 (1.4)
Severe (GCS 3–8)	267 (0.7)
Missing	2,193 (5.6)

AIS, Abbreviated injury severity score; GCS, Glasgow Coma Scale

**Table 3:** Crude outcomes in patients with surgically managed isolated traumatic spinal injuries without spinal cord injury

	Total (*N* = 39,449)
In-hospital mortality, n (%)	554 (1.4)
Any complication, n (%)	2,924 (7.4)
Myocardial infarction, n (%)	94 (0.2)
Cardiac arrest with CPR, n (%)	262 (0.7)
Stroke, n (%)	94 (0.2)
DVT, n (%)	308 (0.8)
Pulmonary embolism, n (%)	171 (0.4)
ARDS, n (%)	134 (0.3)
Acute kidney injury, n (%)	168 (0.4)
Urinary tract infection, n (%)	370 (0.9)
Pneumonia, n (%)	354 (0.9)
Surgical site infection, n (%)	97 (0.2)
Sepsis, n (%)	130 (0.3)
Decubitus ulcer, n (%)	222 (0.6)
Unplanned intubation, n (%)	746 (1.9)
Unplanned admission to the OR, n (%)	276 (0.7)
Unplanned admission to the ICU, n (%)	1,063 (2.7)
Failure-to-rescue, n (%)	349 (0.9)

DVT, Deep vein thrombosis; ARDS, Acute respiratory distress syndrome

When predicting adverse outcomes, the 5-mFI and 11-mFI outperformed all other frailty scores when predicting in-hospital mortality [5-mFI AUC (95% CI): 0.73 (0.71–0.75)] [11-mFI AUC (95% CI): 0.73 (0.71–0.75)], any complication [5-mFI AUC (95% CI): 0.65 (0.64–0.66)] [11-mFI AUC (95% CI): 0.65 (0.64–0.66)], cardiovascular complications [5-mFI AUC (95% CI): 0.71 (0.69–0.74)] [11-mFI AUC (95% CI): 0.72 (0.69–0.74)], infection [5-mFI AUC (95% CI): 0.64 (0.62–0.65)] [11-mFI AUC (95% CI): 0.64 (0.62–0.66)], and FTR [5-mFI AUC (95% CI): 0.75 (0.73–0.78)] [11-mFI AUC (95% CI): 0.75 (0.73–0.78)]. The HFRS outperformed the other scores when predicting respiratory complications [AUC (95% CI): 0.66 (0.64–0.69)]. All scores struggled to predict venous thromboembolisms (Table 4).

**Table 4:** Predictive ability of frailty scores for adverse outcomes in patients with surgically managed isolated traumatic spinal injuries without spinal cord injury

Outcome	AUC (95% CI)	Sensitivity (95% CI)	Specificity (95% CI)	Accuracy (95% CI)	*P*-value for difference in AUCs*
**In-hospital mortality**					
OFS	0.70 (0.67–0.72)	0.52 (0.48–0.56)	0.86 (0.80–0.88)	0.86 (0.85–0.86)	Reference
HFRS	0.65 (0.63–0.67)	0.81 (0.77–0.84)	0.47 (0.40–0.49)	0.47 (0.47–0.48)	0.003
11-mFI	0.73 (0.71–0.75)	0.83 (0.79–0.86)	0.54 (0.46–0.57)	0.55 (0.54–0.55)	0.015
5-mFI	0.73 (0.71–0.75)	0.82 (0.79–0.85)	0.54 (0.46–0.57)	0.55 (0.54–0.55)	0.015
JHFI	0.52 (0.51–0.53)	0.11 (0.08–0.13)	0.93 (0.90–0.94)	0.92 (0.92–0.92)	< 0.001
**Any complication**					
OFS	0.59 (0.58–0.60)	0.31 (0.29–0.33)	0.87 (0.85–0.88)	0.83 (0.83–0.83)	Reference
HFRS	0.61 (0.60–0.62)	0.71 (0.69–0.72)	0.48 (0.45–0.49)	0.50 (0.49–0.50)	0.004
11-mFI	0.65 (0.64–0.66)	0.70 (0.68–0.72)	0.56 (0.53–0.57)	0.57 (0.56–0.57)	< 0.001
5-mFI	0.65 (0.64–0.66)	0.70 (0.68–0.71)	0.56 (0.53–0.57)	0.57 (0.56–0.57)	< 0.001
JHFI	0.52 (0.52–0.53)	0.11 (0.10–0.12)	0.93 (0.92–0.94)	0.87 (0.87–0.87)	< 0.001
**Cardiovascular complication**					
OFS	0.63 (0.61–0.65)	0.40 (0.35–0.44)	0.86 (0.80–0.88)	0.85 (0.85–0.86)	Reference
HFRS	0.64 (0.61–0.66)	0.78 (0.74–0.82)	0.47 (0.39–0.49)	0.47 (0.47–0.48)	0.672
11-mFI	0.72 (0.69–0.74)	0.81 (0.78–0.85)	0.54 (0.45–0.57)	0.54 (0.54–0.55)	< 0.001
5-mFI	0.71 (0.69–0.74)	0.81 (0.77–0.84)	0.54 (0.45–0.57)	0.55 (0.54–0.55)	< 0.001
JHFI	0.53 (0.51–0.54)	0.12 (0.09–0.15)	0.93 (0.90–0.94)	0.92 (0.92–0.92)	< 0.001
**Venous thromboembolism**					
OFS	0.56 (0.54–0.58)	0.25 (0.21–0.29)	0.86 (0.82–0.89)	0.85 (0.85–0.86)	Reference
HFRS	0.58 (0.55–0.60)	0.49 (0.44–0.53)	0.65 (0.59–0.68)	0.65 (0.64–0.65)	0.200
11-mFI	0.59 (0.56–0.61)	0.60 (0.56–0.65)	0.54 (0.48–0.58)	0.54 (0.54–0.55)	0.033
5-mFI	0.59 (0.56–0.61)	0.59 (0.54–0.63)	0.55 (0.50–0.59)	0.55 (0.55–0.56)	0.036
JHFI	0.51 (0.50–0.53)	0.09 (0.07–0.12)	0.93 (0.90–0.95)	0.92 (0.92–0.92)	0.001
**Respiratory complication**					
OFS	0.59 (0.57–0.61)	0.32 (0.28–0.36)	0.86 (0.81–0.88)	0.85 (0.85–0.86)	Reference
HFRS	0.66 (0.64–0.69)	0.58 (0.54–0.63)	0.71 (0.65–0.73)	0.71 (0.70–0.71)	< 0.001
11-mFI	0.64 (0.61–0.66)	0.70 (0.66–0.74)	0.54 (0.47–0.57)	0.54 (0.54–0.55)	< 0.001
5-mFI	0.64 (0.61–0.66)	0.69 (0.65–0.74)	0.54 (0.48–0.57)	0.54 (0.54–0.55)	< 0.001
JHFI	0.54 (0.52–0.56)	0.15 (0.12–0.19)	0.93 (0.90–0.94)	0.92 (0.92–0.92)	< 0.001
**Infection**					
OFS	0.59 (0.57–0.60)	0.31 (0.28–0.35)	0.86 (0.82–0.88)	0.85 (0.85–0.85)	Reference
HFRS	0.62 (0.60–0.64)	0.51 (0.47–0.54)	0.71 (0.67–0.72)	0.71 (0.70–0.71)	0.004
11-mFI	0.64 (0.62–0.66)	0.70 (0.67–0.73)	0.54 (0.50–0.57)	0.55 (0.54–0.55)	< 0.001
5-mFI	0.64 (0.62–0.65)	0.70 (0.66–0.72)	0.55 (0.50–0.57)	0.55 (0.54–0.55)	< 0.001
JHFI	0.53 (0.52–0.54)	0.13 (0.11–0.15)	0.93 (0.91–0.94)	0.91 (0.91–0.92)	< 0.001
**Failure-to-rescue**					
OFS	0.70 (0.67–0.73)	0.54 (0.48–0.58)	0.86 (0.77–0.87)	0.86 (0.85–0.86)	Reference
HFRS	0.66 (0.63–0.68)	0.84 (0.79–0.87)	0.47 (0.38–0.49)	0.47 (0.47–0.48)	0.027
11-mFI	0.75 (0.73–0.78)	0.87 (0.83–0.90)	0.54 (0.42–0.57)	0.54 (0.54–0.55)	< 0.001
5-mFI	0.75 (0.73–0.78)	0.86 (0.82–0.89)	0.54 (0.43–0.57)	0.55 (0.54–0.55)	< 0.001
JHFI	0.51 (0.50–0.53)	0.10 (0.07–0.13)	0.93 (0.90–0.95)	0.92 (0.92–0.92)	< 0.001

*All *p*-values are adjusted using the Holm-Bonferroni method

OFS, Orthopedic Frailty Score; HFRS, Hospital Frailty Risk Score; 11-mFI, 11-factor modified Frailty Index; 5-mFI, 5-factor modified Frailty Index; JHFI, Johns Hopkins Frailty Indicator

Among the 14,257 geriatric patients, however, the OFS demonstrated the highest predictive ability for in-hospital mortality [AUC (95% CI): 0.65 (0.62–0.67)]. The OFS also performed on the same level [AUC (95% CI): 0.64 (0.61–0.67)] as both the 5-mFI [AUC (95% CI): 0.63 (0.60–0.66)] and the 11-mFI [AUC (95% CI): 0.63 (0.60–0.66)] when predicting FTR in this population. All frailty scores struggled to predict complications in geriatric patients with surgically managed isolated TSI without SCI (Table 5).

**Table 5:** Predictive ability of frailty scores for adverse outcomes in geriatric patients with surgically managed isolated traumatic spinal injuries without spinal cord injury

Outcome	AUC (95% CI)	Sensitivity (95% CI)	Specificity (95% CI)	Accuracy (95% CI)	*P*-value for difference in AUCs*
**In-hospital mortality**					
OFS	0.65 (0.62–0.67)	0.59 (0.55–0.64)	0.68 (0.61–0.71)	0.67 (0.67–0.68)	Reference
HFRS	0.57 (0.55–0.60)	0.43 (0.38–0.47)	0.68 (0.63–0.71)	0.67 (0.66–0.68)	< 0.001
11-mFI	0.61 (0.59–0.64)	0.59 (0.54–0.64)	0.59 (0.54–0.62)	0.59 (0.58–0.60)	0.046
5-mFI	0.62 (0.59–0.64)	0.59 (0.54–0.64)	0.60 (0.55–0.63)	0.60 (0.59–0.61)	0.046
JHFI	0.51 (0.49–0.52)	0.90 (0.88–0.93)	0.11 (0.09–0.14)	0.14 (0.13–0.14)	< 0.001
**Any complication**					
OFS	0.56 (0.55–0.58)	0.44 (0.42–0.47)	0.69 (0.66–0.70)	0.65 (0.65–0.66)	Reference
HFRS	0.56 (0.54–0.57)	0.61 (0.59–0.64)	0.48 (0.45–0.50)	0.49 (0.49–0.50)	1.00
11-mFI	0.57 (0.56–0.58)	0.51 (0.49–0.54)	0.60 (0.57–0.62)	0.59 (0.58–0.60)	1.00
5-mFI	0.57 (0.55–0.58)	0.50 (0.48–0.52)	0.61 (0.58–0.63)	0.60 (0.59–0.60)	1.00
JHFI	0.51 (0.50–0.52)	0.13 (0.12–0.15)	0.89 (0.88–0.91)	0.80 (0.79–0.80)	< 0.001
**Cardiovascular complication**					
OFS	0.57 (0.54–0.59)	0.46 (0.40–0.51)	0.67 (0.61–0.71)	0.67 (0.66–0.67)	Reference
HFRS	0.56 (0.53–0.59)	0.45 (0.39–0.50)	0.68 (0.59–0.70)	0.67 (0.67–0.68)	1.00
11-mFI	0.58 (0.55–0.61)	0.52 (0.47–0.57)	0.59 (0.53–0.63)	0.59 (0.58–0.59)	1.00
5-mFI	0.57 (0.54–0.60)	0.51 (0.46–0.56)	0.60 (0.54–0.64)	0.60 (0.59–0.60)	1.00
JHFI	0.51 (0.49–0.53)	0.13 (0.09–0.16)	0.89 (0.86–0.91)	0.87 (0.87–0.88)	0.002
**Venous thromboembolism**					
OFS	0.55 (0.52–0.58)	0.34 (0.28–0.40)	0.75 (0.69–0.79)	0.74 (0.74–0.75)	Reference
HFRS	0.54 (0.50–0.58)	0.56 (0.49–0.62)	0.52 (0.45–0.57)	0.52 (0.52–0.53)	1.00
11-mFI	0.56 (0.52–0.59)	0.68 (0.62–0.74)	0.40 (0.34–0.46)	0.41 (0.40–0.42)	1.00
5-mFI	0.56 (0.53–0.60)	0.69 (0.63–0.75)	0.41 (0.35–0.46)	0.41 (0.40–0.42)	1.00
JHFI	0.51 (0.49–0.53)	0.12 (0.08–0.17)	0.89 (0.85–0.92)	0.88 (0.87–0.88)	0.136
**Respiratory complication**					
OFS	0.56 (0.53–0.59)	0.44 (0.38–0.49)	0.68 (0.62–0.73)	0.68 (0.67–0.69)	Reference
HFRS	0.61 (0.58–0.65)	0.65 (0.59–0.70)	0.58 (0.49–0.61)	0.58 (0.57–0.59)	0.058
11-mFI	0.55 (0.52–0.58)	0.48 (0.42–0.54)	0.59 (0.54–0.64)	0.59 (0.58–0.60)	0.528
5-mFI	0.54 (0.50–0.57)	0.47 (0.41–0.52)	0.60 (0.54–0.65)	0.59 (0.59–0.60)	0.509
JHFI	0.53 (0.51–0.56)	0.18 (0.13–0.22)	0.89 (0.84–0.91)	0.88 (0.87–0.88)	0.509
**Infection**					
OFS	0.55 (0.53–0.58)	0.42 (0.38–0.46)	0.68 (0.64–0.72)	0.68 (0.67–0.68)	Reference
HFRS	0.58 (0.56–0.61)	0.59 (0.54–0.63)	0.58 (0.51–0.61)	0.58 (0.57–0.59)	0.263
11-mFI	0.54 (0.51–0.56)	0.83 (0.80–0.86)	0.21 (0.18–0.24)	0.23 (0.23–0.24)	0.263
5-mFI	0.53 (0.51–0.56)	0.83 (0.79–0.86)	0.22 (0.18–0.25)	0.24 (0.23–0.25)	0.263
JHFI	0.53 (0.51–0.54)	0.16 (0.13–0.19)	0.89 (0.86–0.91)	0.86 (0.86–0.87)	0.145
**Failure-to-rescue**					
OFS	0.64 (0.61–0.67)	0.59 (0.53–0.65)	0.67 (0.59–0.71)	0.67 (0.66–0.68)	Reference
HFRS	0.57 (0.54–0.60)	0.85 (0.81–0.89)	0.26 (0.20–0.29)	0.27 (0.26–0.28)	0.003
11-mFI	0.63 (0.60–0.66)	0.62 (0.57–0.68)	0.59 (0.53–0.63)	0.59 (0.58–0.60)	1.00
5-mFI	0.63 (0.60–0.66)	0.62 (0.57–0.68)	0.60 (0.53–0.64)	0.60 (0.59–0.61)	1.00
JHFI	0.51 (0.49–0.52)	0.90 (0.87–0.93)	0.11 (0.08–0.14)	0.13 (0.12–0.13)	< 0.001

*All *p*-values are adjusted using the Holm-Bonferroni method

OFS, Orthopedic Frailty Score; HFRS, Hospital Frailty Risk Score; 11-mFI, 11-factor modified Frailty Index; 5-mFI, 5-factor modified Frailty Index; JHFI, Johns Hopkins Frailty Indicator

Among the 9616 patients who were injured due to a ground-level fall, the OFS [AUC (95% CI): 0.66 (0.63–0.69)] performed on par with the 5-mFI [AUC (95% CI): 0.63 (0.60–0.66)] and 11-mFI [AUC (95% CI): 0.63 (0.60–0.66)] when predicting in-hospital mortality. This was also the case when predicting FTR in this population [AUC (95% CI), OFS vs. 5-mFI vs. 11-mFI: 0.67 (0.64–0.71) vs. 0.65 (0.61–0.68) vs. 0.65 (0.61–0.69)]. All frailty scores struggled to predict complications, apart from the HFRS when predicting respiratory complications [0.65 (0.60–0.69)] (Supplemental Table 1).

## Discussion

Among the frailty scores included in the current study, only the 5-mFI, 11-mFI, and OFS were able to achieve an acceptable predictive ability for any of the adverse outcomes included in the analysis [24]. Of these, the 5-mFI and 11-mFI tended to perform the best. On the other hand, the OFS outperformed or performed on par with these frailty scores among the geriatric patient population and those injured in a ground-level fall. This was the case despite the 5-mFI and OFS both being the two simplest scores to calculate among those included in the investigation.

Results of previous studies have varied. In a study by Shakil et al. on adult patients with complete cervical SCI registered in TQIP, they calculated a similar AUC (95% CI) for the 5-mFI of 0.74 (0.72–0.76) when predicting in-hospital mortality. However, this model also included sex, ethnicity, insurance type, mechanism of injury, presenting GCS, presence of shock, whether they underwent surgery, hospital ACS verification level, teaching status, hospital size, and year of injury, making it difficult to evaluate the predictive value of the 5-mFI alone [25]. On the other hand, in a different investigation by Bowers et al. investigating the predictive ability of the 5-mFI in surgically managed spine trauma patients, it only achieved an AUC (95% CI) of 0.62 (0.61–0.64) for 30-day mortality as well as Clavien-Dindo grade IV complications [26]. Similarly, Conlon et al. performed a study on TSI patients where they found that the 5-mFI exhibited an AUC (95% CI) of 0.62 (0.61–0.63) for 30-day mortality and and an AUC between 0.60 and 0.65 for both major complications and Clavien-Dindo grade IV complications [27]. 

The observation that simpler frailty scores, such as the 5-mFI and OFS, performed on par with or outperformed more complicated scores, such as the 11-mFI and HFRS, bears highlighting. Firstly, simplified scores provide an efficient assessment process that is both immediate and straightforward to implement in clinical settings, enhancing usability and thereby rendering them more practical for routine utilization, particularly in demanding settings such as trauma centers [28]. Moreover, in the context of research, simpler frailty scores could more easily facilitate larger-scale studies, as these scores demand less detailed patient data, allowing for application across broader patient populations, as evidenced by the high prevalence of studies using simpler scores [11]. However, while simpler scores are generally sufficient for most surgically managed TSI patients, the prediction of specific outcomes may benefit from more detailed frailty assessments. For instance, the HFRS demonstrated the highest predictive ability for respiratory complications, performing at the same level as the 5-mFI (*p* = 0.120) and 11-mFI (*p* = 0.181) in the total cohort and outperforming them in the geriatric cohort (*p* = 0.002 and *p* = 0.007). Nevertheless, the absolute difference in AUC between the HFRS and 5-mFI may not justify opting for a score with 109 weighted variables over one with just 5 binary variables.

The primary benefit of frailty scores is that they offer a systematic method for evaluating a patient’s physiological reserve and susceptibility to negative outcomes [29, 30]. By risk stratifying patients, healthcare personnel can identify those requiring enhanced monitoring and foresee the necessity for more intensive postoperative care, allowing for more effective allocation of resources. Furthermore, frailty assessments may facilitate personalized treatment decisions. For instance, frail patients might in some cases benefit from more conservative surgical options or additional supportive measures, such as “prehabilitation”, to minimize associated risks [31–34]. This individualized approach helps balance the potential benefits and drawbacks of surgical intervention in more vulnerable patients, thereby enhancing patient-centered care. Finally, frailty assessments provide evidence-based estimates of potential outcomes, including recovery time, complications, and long-term prognosis [3, 6–10]. This information supports shared decision-making by enabling patients to make well-informed choices and set realistic expectations regarding the risks and benefits of surgery.

While the OFS, 5-mFI, and 11-mFI demonstrated some ability to predict in-hospital mortality and FTR, predicting complications proved to be more challenging. This limitation was particularly pronounced in the subgroup analyses, where nearly all frailty scores showed minimal predictive capability for complications. This may reflect the influence of factors beyond frailty that play a more significant role in determining in-hospital complications. Although frailty was identified in a recent publication as one of the top ten predictors of complications in TSI patients without SCI, other factors such as age, cervical spine injury, the need for cervical spine surgery, the Revised Cardiac Risk Index, and alcohol use disorder were all found to have greater predictive importance in this population [15]. 

A key advantage of this study is the use of a large and multi-institutional dataset, thereby increasing the generalizability of the current findings. This study also benefits from the inclusion of a wide range of validated frailty scores, enabling comparison across various methods for assessing frailty that differ in complexity. Nevertheless, several limitations must be recognized. Firstly, the study is limited by its retrospective design, which presents intrinsic constraints such as potential for selection bias and misclassification. Despite measures put in place to mitigate the risk of data errors [35], the risk of non-differential misclassification always remains in a dataset of this size, which could bias the results towards the null. Secondly, while many frailty scores were included in the analysis, the study remained constrained by the variables available in TQIP. Accordingly, several other established frailty scores could not be included, such as the Clinical Frailty Scale, Edmonton Frail Scale, frailty phenotype, and Frailty Index. Furthermore, this study was not able to evaluate the included scores’ predictive ability for additional outcomes of interest such as readmission rates, quality of life, long-term mortality, and long-term functional outcomes.

## Conclusion

Simpler scores like the 5-factor modified Frailty Index and Orthopedic Frailty Score outperform or perform on par with more complicated frailty scores when predicting mortality, complications, and failure-to-rescue in surgically managed isolated traumatic spinal injury patients without spinal cord injury, particularly among geriatric patients and those injured in a ground-level fall.

## Electronic supplementary material

Below is the link to the electronic supplementary material.

Supplementary Material 1

## Data Availability

No datasets were generated or analysed during the current study.

## Declarations

### Congress presentation

The abstract of this paper has been accepted for presentation at the 24th European Congress of Trauma and Emergency Surgery in Aachen, Germany, between 13 and 15 April 2025.
