# Supplementary material for: Predictive ability of frailty scores in surgically managed patients with traumatic spinal injuries: a TQIP analysis
Source: Eur J Trauma Emerg Surg. 2025 Mar 4;51(1):126. doi: 10.1007/s00068-025-02775-0 (PMC11880054; doi:10.1007/s00068-025-02775-0)
Supplement: Supplementary file 1 — Supplementary Material 1 [file 68_2025_2775_MOESM1_ESM.docx]

| **Supplemental table 1.** Predictive ability of frailty scores for adverse outcomes in patients with surgically managed isolated traumatic spinal injuries without spinal cord injury caused by a ground-level fall | | | | | |
| --- | --- | --- | --- | --- | --- |
| **Outcome** | **AUC**  **(95% CI)** | **Sensitivity**  **(95% CI)** | **Specificity**  **(95% CI)** | **Accuracy**  **(95% CI)** | **P-value for difference in AUCs*** |
| **In-hospital mortality** |  |  |  |  |  |
| OFS | 0.66 (0.63-0.69) | 0.64 (0.59-0.70) | 0.66 (0.57-0.69) | 0.66 (0.65-0.66) | Reference |
| HFRS | 0.54 (0.50-0.57) | 0.33 (0.26-0.38) | 0.75 (0.68-0.78) | 0.74 (0.73-0.75) | <0.001 |
| 11-mFI | 0.63 (0.60-0.66) | 0.61 (0.56-0.66) | 0.59 (0.53-0.63) | 0.59 (0.58-0.60) | 0.148 |
| 5-mFI | 0.63 (0.60-0.66) | 0.61 (0.56-0.66) | 0.59 (0.54-0.63) | 0.60 (0.59-0.61) | 0.148 |
| JHFI | 0.51 (0.50-0.52) | 0.04 (0.02-0.06) | 0.98 (0.96-0.99) | 0.96 (0.95-0.96) | <0.001 |
| **Any complication** |  |  |  |  |  |
| OFS | 0.57 (0.55-0.58) | 0.47 (0.44-0.50) | 0.66 (0.63-0.69) | 0.64 (0.63-0.65) | Reference |
| HFRS | 0.55 (0.53-0.57) | 0.59 (0.55-0.62) | 0.51 (0.47-0.53) | 0.52 (0.51-0.53) | 0.194 |
| 11-mFI | 0.59 (0.57-0.61) | 0.54 (0.51-0.57) | 0.60 (0.56-0.62) | 0.59 (0.58-0.60) | 0.035 |
| 5-mFI | 0.59 (0.57-0.60) | 0.53 (0.50-0.56) | 0.60 (0.57-0.63) | 0.60 (0.59-0.61) | 0.035 |
| JHFI | 0.51 (0.50-0.51) | 0.03 (0.02-0.04) | 0.98 (0.98-0.99) | 0.88 (0.87-0.88) | <0.001 |
| **Cardiovascular complication** |  |  |  |  |  |
| OFS | 0.60 (0.57-0.64) | 0.55 (0.48-0.62) | 0.65 (0.57-0.69) | 0.65 (0.64-0.66) | Reference |
| HFRS | 0.55 (0.51-0.59) | 0.38 (0.31-0.45) | 0.76 (0.65-0.79) | 0.75 (0.74-0.76) | 0.248 |
| 11-mFI | 0.61 (0.57-0.65) | 0.57 (0.50-0.64) | 0.58 (0.51-0.64) | 0.58 (0.57-0.59) | 1.000 |
| 5-mFI | 0.61 (0.57-0.64) | 0.56 (0.50-0.63) | 0.60 (0.52-0.65) | 0.59 (0.58-0.60) | 1.000 |
| JHFI | 0.51 (0.50-0.52) | 0.03 (0.01-0.05) | 0.98 (0.96-0.99) | 0.96 (0.96-0.97) | <0.001 |
| **Venous thromboembolism** |  |  |  |  |  |
| OFS | 0.55 (0.50-0.59) | 0.35 (0.27-0.42) | 0.74 (0.65-0.79) | 0.73 (0.72-0.74) | Reference |
| HFRS | 0.53 (0.48-0.58) | 0.60 (0.51-0.66) | 0.50 (0.40-0.54) | 0.50 (0.49-0.51) | 1.000 |
| 11-mFI | 0.56 (0.52-0.61) | 0.43 (0.35-0.51) | 0.66 (0.58-0.72) | 0.65 (0.64-0.66) | 1.000 |
| 5-mFI | 0.57 (0.52-0.61) | 0.44 (0.36-0.52) | 0.65 (0.58-0.72) | 0.65 (0.64-0.66) | 1.000 |
| JHFI | 0.51 (0.49-0.52) | 0.03 (0.01-0.06) | 0.98 (0.96-0.99) | 0.97 (0.97-0.97) | 0.279 |
| **Respiratory complication** |  |  |  |  |  |
| OFS | 0.57 (0.53-0.62) | 0.40 (0.31-0.47) | 0.74 (0.64-0.79) | 0.73 (0.73-0.74) | Reference |
| HFRS | 0.65 (0.60-0.69) | 0.79 (0.72-0.86) | 0.54 (0.37-0.58) | 0.55 (0.54-0.56) | 0.043 |
| 11-mFI | 0.57 (0.52-0.61) | 0.52 (0.44-0.61) | 0.58 (0.50-0.65) | 0.58 (0.57-0.59) | 1.000 |
| 5-mFI | 0.57 (0.52-0.62) | 0.51 (0.43-0.60) | 0.59 (0.51-0.65) | 0.59 (0.58-0.60) | 1.000 |
| JHFI | 0.50 (0.50-0.51) | 0.99 (0.98-1.00) | 0.02 (0.00-0.03) | 0.03 (0.03-0.03) | 0.011 |
| **Infection** |  |  |  |  |  |
| OFS | 0.56 (0.52-0.59) | 0.44 (0.38-0.50) | 0.66 (0.60-0.71) | 0.66 (0.65-0.67) | Reference |
| HFRS | 0.59 (0.56-0.63) | 0.71 (0.65-0.77) | 0.54 (0.41-0.57) | 0.55 (0.54-0.56) | 0.365 |
| 11-mFI | 0.57 (0.53-0.60) | 0.87 (0.82-0.91) | 0.24 (0.18-0.28) | 0.26 (0.25-0.26) | 0.712 |
| 5-mFI | 0.57 (0.54-0.61) | 0.87 (0.82-0.91) | 0.24 (0.18-0.28) | 0.26 (0.25-0.27) | 0.712 |
| JHFI | 0.50 (0.49-0.51) | 0.02 (0.00-0.04) | 0.98 (0.97-0.99) | 0.96 (0.96-0.96) | 0.006 |
| **Failure-to-rescue** |  |  |  |  |  |
| OFS | 0.67 (0.64-0.71) | 0.68 (0.60-0.74) | 0.65 (0.53-0.69) | 0.65 (0.64-0.66) | Reference |
| HFRS | 0.52 (0.48-0.56) | 0.31 (0.23-0.36) | 0.75 (0.66-0.79) | 0.75 (0.74-0.75) | <0.001 |
| 11-mFI | 0.65 (0.61-0.69) | 0.63 (0.56-0.70) | 0.58 (0.51-0.64) | 0.59 (0.58-0.60) | 0.585 |
| 5-mFI | 0.65 (0.61-0.68) | 0.63 (0.56-0.70) | 0.59 (0.52-0.64) | 0.59 (0.58-0.60) | 0.585 |
| JHFI | 0.51 (0.50-0.53) | 0.04 (0.01-0.07) | 0.98 (0.96-0.99) | 0.97 (0.96-0.97) | <0.001 |
| *All p-values are adjusted using the Holm-Bonferroni method  *OFS, Orthopedic Frailty Score; HFRS, Hospital Frailty Risk Score; 11-mFI, 11-factor modified Frailty Index; 5-mFI, 5-factor modified Frailty Index; JHFI, Johns Hopkins Frailty Indicator* | | | | | |
